# Redetermination of {2-[3-(dimethyl­ammonio)propyl­imino­meth­yl]phenol­ato}­dithio­cyanato­zinc(II)

**DOI:** 10.1107/S1600536809009568

**Published:** 2009-03-19

**Authors:** Zhe Hong

**Affiliations:** a College of Chemical Engineering and Materials Science, Liaodong University, Dandong 118003, People’s Republic of China

## Abstract

In comparison with the previous refinement of the title complex, [Zn(NCS)_2_(C_12_H_18_N_2_O)], the present redetermination reveals a different location of the non-carbon attached H atom. Whereas in the previous refinement this H atom was modelled as part of a phenol OH group, the present study indicates a zwitterionic Schiff base ligand with a deprotonated OH group and a protonated tertiary amine group. The Zn(II) atom is four-coordinated by one O and one imine N atoms of the 2-[3-(dimethyl­ammonio)propyl­imino­meth­yl]phenolate Schiff base ligand, and by two N atoms from two thio­cyanate ligands, forming a distorted tetra­hedral geometry. In the crystal structure, adjacent mol­ecules are linked through inter­molecular N—H⋯O hydrogen bonds, forming a chain in the [101] direction.

## Related literature

For a previous refinement of this structure, see: Cai *et al.* (2006 ▶).
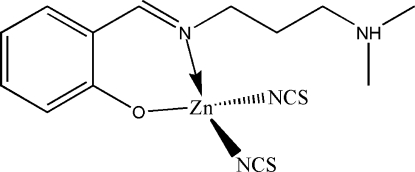


## Experimental

### 

#### Crystal data

[Zn(NCS)_2_(C_12_H_18_N_2_O)]
*M* *_r_* = 387.81
Monoclinic, 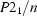

*a* = 9.850 (2) Å
*b* = 14.931 (3) Å
*c* = 12.290 (3) Å
β = 101.450 (2)°
*V* = 1771.5 (7) Å^3^
*Z* = 4
Mo *K*α radiation
μ = 1.63 mm^−1^
*T* = 298 K
0.23 × 0.20 × 0.20 mm

#### Data collection

Bruker SMART CCD area-detector diffractometer
Absorption correction: multi-scan (*SADABS*; Sheldrick, 1996 ▶) *T*_min_ = 0.706, *T*_max_ = 0.737
10464 measured reflections
4067 independent reflections
3048 reflections with *I* > 2σ(*I*)
*R*_int_ = 0.026

#### Refinement

*R*[*F* ^2^ > 2σ(*F* ^2^)] = 0.036
*wR*(*F* ^2^) = 0.089
*S* = 1.03
4067 reflections
205 parameters
1 restraint
H atoms treated by a mixture of independent and constrained refinement
Δρ_max_ = 0.59 e Å^−3^
Δρ_min_ = −0.49 e Å^−3^

### 

Data collection: *SMART* (Bruker, 2002 ▶); cell refinement: *SAINT* (Bruker, 2002 ▶); data reduction: *SAINT*; program(s) used to solve structure: *SHELXS97* (Sheldrick, 2008 ▶); program(s) used to refine structure: *SHELXL97* (Sheldrick, 2008 ▶); molecular graphics: *SHELXTL* (Sheldrick, 2008 ▶); software used to prepare material for publication: *SHELXTL*.

## Supplementary Material

Crystal structure: contains datablocks global, I. DOI: 10.1107/S1600536809009568/er2062sup1.cif

Structure factors: contains datablocks I. DOI: 10.1107/S1600536809009568/er2062Isup2.hkl

Additional supplementary materials:  crystallographic information; 3D view; checkCIF report

## Figures and Tables

**Table 1:** Hydrogen-bond geometry (Å, °)

*D*—H⋯*A*	*D*—H	H⋯*A*	*D*⋯*A*	*D*—H⋯*A*
N2—H2⋯O1^i^	0.891 (10)	1.855 (11)	2.737 (2)	170 (2)

Symmetry code: (i) 
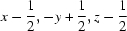
.

**Fig. 1.**
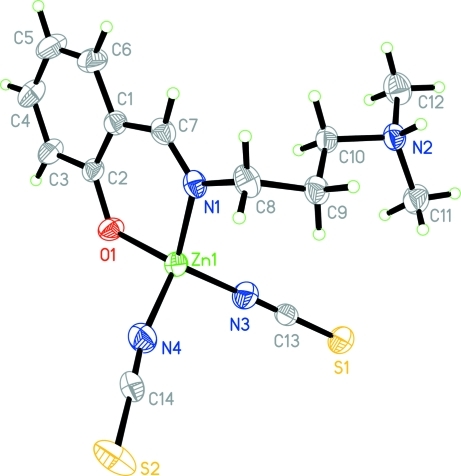
The structure of compound (I), showing 30% probability displacement ellipsoids and the atom-numbering scheme.

**Fig. 2.**
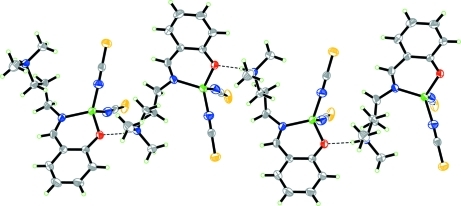
Molecular packing of compound (I), viewed perpendicular to the [101] direction. Intermolecular N—H···O hydrogen bonds are shown as dashed lines.

## supplementary crystallographic information

### Comment

Previously, Cai *et al.* (2006) have reported the crystal structure of the title mononuclear zinc(II) complex, (I), with the non-carbon attached H atom located at the phenolate O atom. The present redetermination of (I) indicates that the H atom should be attached to the amine N atom.

Complex (I) is a mononuclear zinc(II) compound. The Zn atom in (I) is four-coordinated by one O and one imine N atoms of a Schiff base ligand [(3-dimethylammoniopropylimino)methyl]phenolate, and by two N atoms from two thiocyanate ligands, forming a tetrahedral geometry. All the bond lengths and angles are comparable to those observed in the previously reported structure, (II) (Cai *et al.*, 2006). The main difference lies in the positions of the non-carbon attached H atoms. The H2 in (I) is attached to N2, while that in (II) is attached to O1.

In the crystal structure of (I), molecules are linked through intermolecular N—H···O hydrogen bonds (Table 1), forming chains running along the [101] direction (Fig. 2). While that in the crystal structure of (II), a strong hydrogen bond interaction is presented between the phenolic hydroxyl H and the uncoordinated amine N, forming a one-dimensional chain.

### Experimental

Salicylaldehyde (1.0 mmol, 122.1 mg), *N,N*-dimethylpropane-1,3-diamine (1.0 mmol, 102.2 mg), ammonium thiocyanate (2.0 mmol, 152.0 mg) and Zn(CH_3_COO)_2_.2H_2_O (1.0 mmol, 219.5 mg) were dissolved in a methanol solution (30 ml). The mixture was stirred at room temperature for 30 min to give a clear colorless solution. After keeping the solution in air for a few days, colorless block-shaped crystals were formed.

### Refinement

H2 was located from a difference Fourier map and refined isotropically, with N—H distance restrained to 0.90 (1) Å. Other H atoms were placed in idealized positions and constrained to ride on their parent atoms with C—H distances of 0.93–0.97 Å, and with *U*_iso_(H) set to 1.2*U*_eq_(C) and 1.5*U*_eq_(methyl C).

### Crystal data

**Table:**

[Zn(NCS)_2_(C_12_H_18_N_2_O)]	*F*(000) = 800
*M**_r_* = 387.81	*D*_x_ = 1.454 Mg m^−^^3^
Monoclinic, *P*2_1_/*n*	Mo *K*α radiation, λ = 0.71073 Å
Hall symbol: -P 2yn	Cell parameters from 3352 reflections
*a* = 9.850 (2) Å	θ = 2.3–25.5°
*b* = 14.931 (3) Å	µ = 1.63 mm^−^^1^
*c* = 12.290 (3) Å	*T* = 298 K
β = 101.450 (2)°	Block, colorless
*V* = 1771.5 (7) Å^3^	0.23 × 0.20 × 0.20 mm
*Z* = 4	

### Data collection

**Table:**

Bruker SMART CCD area-detector diffractometer	4067 independent reflections
Radiation source: fine-focus sealed tube	3048 reflections with *I* > 2σ(*I*)
graphite	*R*_int_ = 0.026
ω scans	θ_max_ = 27.5°, θ_min_ = 2.2°
Absorption correction: multi-scan (*SADABS*; Sheldrick, 1996)	*h* = −11→12
*T*_min_ = 0.706, *T*_max_ = 0.737	*k* = −19→15
10464 measured reflections	*l* = −15→15

### Refinement

**Table:**

Refinement on *F*^2^	Primary atom site location: structure-invariant direct methods
Least-squares matrix: full	Secondary atom site location: difference Fourier map
*R*[*F*^2^ > 2σ(*F*^2^)] = 0.036	Hydrogen site location: inferred from neighbouring sites
*wR*(*F*^2^) = 0.089	H atoms treated by a mixture of independent and constrained refinement
*S* = 1.03	*w* = 1/[σ^2^(*F*_o_^2^) + (0.039*P*)^2^ + 0.5*P*] where *P* = (*F*_o_^2^ + 2*F*_c_^2^)/3
4067 reflections	(Δ/σ)_max_ < 0.001
205 parameters	Δρ_max_ = 0.59 e Å^−^^3^
1 restraint	Δρ_min_ = −0.49 e Å^−^^3^

### Special details

**Table:**

Geometry. All e.s.d.'s (except the e.s.d. in the dihedral angle between two l.s. planes) are estimated using the full covariance matrix. The cell e.s.d.'s are taken into account individually in the estimation of e.s.d.'s in distances, angles and torsion angles; correlations between e.s.d.'s in cell parameters are only used when they are defined by crystal symmetry. An approximate (isotropic) treatment of cell e.s.d.'s is used for estimating e.s.d.'s involving l.s. planes.
Refinement. Refinement of *F*^2^ against ALL reflections. The weighted *R*-factor *wR* and goodness of fit *S* are based on *F*^2^, conventional *R*-factors *R* are based on *F*, with *F* set to zero for negative *F*^2^. The threshold expression of *F*^2^ > σ(*F*^2^) is used only for calculating *R*-factors(gt) *etc*. and is not relevant to the choice of reflections for refinement. *R*-factors based on *F*^2^ are statistically about twice as large as those based on *F*, and *R*- factors based on ALL data will be even larger.

### Fractional atomic coordinates and isotropic or equivalent isotropic displacement parameters (Å^2^)

**Table:**

	*x*	*y*	*z*	*U*_iso_*/*U*_eq_	
Zn1	0.85499 (3)	0.192204 (19)	0.33421 (2)	0.04580 (11)	
S1	1.05230 (8)	0.47887 (5)	0.33292 (6)	0.0647 (2)	
S2	0.62695 (9)	0.21786 (8)	0.63228 (6)	0.0849 (3)	
O1	0.98390 (17)	0.09278 (11)	0.33954 (13)	0.0506 (4)	
N1	0.72661 (19)	0.15862 (14)	0.19440 (16)	0.0444 (4)	
N2	0.7435 (2)	0.38001 (14)	−0.03594 (16)	0.0473 (5)	
N3	0.9446 (3)	0.30791 (16)	0.3333 (2)	0.0607 (6)	
N4	0.7670 (3)	0.19029 (16)	0.4625 (2)	0.0660 (6)	
C1	0.8647 (2)	0.02717 (16)	0.16587 (19)	0.0448 (5)	
C2	0.9731 (2)	0.03075 (15)	0.26025 (19)	0.0431 (5)	
C3	1.0747 (3)	−0.03634 (17)	0.2697 (2)	0.0558 (7)	
H3	1.1456	−0.0368	0.3321	0.067*	
C4	1.0736 (3)	−0.10103 (18)	0.1908 (3)	0.0637 (7)	
H4	1.1444	−0.1432	0.1997	0.076*	
C5	0.9690 (3)	−0.1042 (2)	0.0987 (3)	0.0708 (8)	
H5	0.9685	−0.1479	0.0446	0.085*	
C6	0.8654 (3)	−0.0416 (2)	0.0880 (2)	0.0650 (8)	
H6	0.7927	−0.0449	0.0269	0.078*	
C7	0.7495 (2)	0.08861 (17)	0.14064 (19)	0.0483 (6)	
H7	0.6836	0.0757	0.0773	0.058*	
C8	0.6009 (3)	0.21127 (19)	0.1489 (2)	0.0556 (7)	
H8A	0.5460	0.2186	0.2057	0.067*	
H8B	0.5454	0.1791	0.0872	0.067*	
C9	0.6387 (3)	0.30309 (17)	0.1095 (2)	0.0518 (6)	
H9A	0.5547	0.3354	0.0777	0.062*	
H9B	0.6877	0.3372	0.1724	0.062*	
C10	0.7288 (3)	0.29423 (15)	0.0237 (2)	0.0464 (6)	
H10A	0.8200	0.2737	0.0600	0.056*	
H10B	0.6892	0.2491	−0.0302	0.056*	
C11	0.7994 (3)	0.45391 (19)	0.0407 (2)	0.0682 (8)	
H11A	0.8824	0.4343	0.0897	0.102*	
H11B	0.7319	0.4708	0.0836	0.102*	
H11C	0.8199	0.5045	−0.0014	0.102*	
C12	0.8300 (3)	0.3647 (2)	−0.1207 (2)	0.0707 (8)	
H12A	0.8385	0.4196	−0.1594	0.106*	
H12B	0.7871	0.3201	−0.1726	0.106*	
H12C	0.9203	0.3445	−0.0847	0.106*	
C13	0.9883 (2)	0.37954 (18)	0.33335 (18)	0.0448 (5)	
C14	0.7095 (3)	0.20301 (17)	0.5332 (2)	0.0497 (6)	
H2	0.6584 (14)	0.3946 (16)	−0.0715 (18)	0.052 (7)*	

### Atomic displacement parameters (Å^2^)

**Table:**

	*U*^11^	*U*^22^	*U*^33^	*U*^12^	*U*^13^	*U*^23^
Zn1	0.04936 (18)	0.04294 (18)	0.04378 (16)	0.00497 (12)	0.00605 (12)	0.00177 (12)
S1	0.0756 (5)	0.0494 (4)	0.0688 (4)	−0.0091 (3)	0.0139 (4)	−0.0034 (3)
S2	0.0603 (5)	0.1489 (9)	0.0473 (4)	0.0125 (5)	0.0153 (3)	0.0184 (5)
O1	0.0508 (9)	0.0474 (10)	0.0470 (9)	0.0101 (8)	−0.0062 (7)	−0.0092 (7)
N1	0.0383 (10)	0.0463 (12)	0.0463 (10)	−0.0015 (9)	0.0030 (8)	0.0095 (9)
N2	0.0446 (12)	0.0429 (12)	0.0504 (11)	0.0003 (9)	−0.0003 (9)	0.0070 (9)
N3	0.0709 (15)	0.0498 (14)	0.0631 (14)	−0.0065 (12)	0.0173 (12)	−0.0009 (11)
N4	0.0793 (17)	0.0650 (16)	0.0590 (14)	0.0083 (12)	0.0263 (13)	0.0087 (12)
C1	0.0465 (13)	0.0402 (13)	0.0451 (12)	−0.0050 (10)	0.0026 (10)	0.0010 (10)
C2	0.0444 (13)	0.0360 (12)	0.0466 (13)	−0.0043 (10)	0.0035 (10)	−0.0007 (10)
C3	0.0513 (15)	0.0421 (15)	0.0670 (16)	0.0036 (11)	−0.0050 (13)	−0.0076 (12)
C4	0.0609 (17)	0.0443 (16)	0.085 (2)	0.0042 (12)	0.0124 (15)	−0.0132 (14)
C5	0.081 (2)	0.0522 (18)	0.0759 (19)	−0.0022 (15)	0.0075 (17)	−0.0234 (15)
C6	0.0702 (19)	0.0597 (18)	0.0571 (16)	−0.0072 (15)	−0.0063 (14)	−0.0140 (14)
C7	0.0447 (13)	0.0534 (16)	0.0425 (12)	−0.0083 (11)	−0.0022 (10)	0.0070 (11)
C8	0.0402 (13)	0.0674 (18)	0.0574 (15)	0.0064 (12)	0.0050 (11)	0.0153 (13)
C9	0.0471 (14)	0.0537 (16)	0.0516 (14)	0.0128 (11)	0.0025 (11)	0.0081 (12)
C10	0.0467 (13)	0.0396 (14)	0.0503 (13)	0.0033 (10)	0.0035 (11)	0.0054 (10)
C11	0.080 (2)	0.0461 (16)	0.0701 (18)	−0.0098 (14)	−0.0063 (15)	0.0001 (14)
C12	0.0704 (19)	0.076 (2)	0.0696 (18)	0.0019 (16)	0.0240 (15)	0.0154 (16)
C13	0.0457 (13)	0.0513 (15)	0.0373 (11)	0.0069 (11)	0.0077 (10)	0.0003 (11)
C14	0.0475 (14)	0.0519 (16)	0.0472 (13)	−0.0006 (11)	0.0035 (11)	0.0143 (12)

### Geometric parameters (Å, °)

**Table:**

Zn1—O1	1.946 (2)	C4—C5	1.372 (4)
Zn1—N1	1.985 (2)	C4—H4	0.9300
Zn1—N3	1.941 (2)	C5—C6	1.371 (4)
Zn1—N4	1.945 (2)	C5—H5	0.9300
S1—C13	1.612 (3)	C6—H6	0.9300
S2—C14	1.608 (3)	C7—H7	0.9300
O1—C2	1.333 (3)	C8—C9	1.524 (4)
N1—C7	1.280 (3)	C8—H8A	0.9700
N1—C8	1.479 (3)	C8—H8B	0.9700
N2—C11	1.484 (3)	C9—C10	1.513 (4)
N2—C12	1.489 (3)	C9—H9A	0.9700
N2—C10	1.497 (3)	C9—H9B	0.9700
N2—H2	0.891 (10)	C10—H10A	0.9700
N3—C13	1.153 (3)	C10—H10B	0.9700
N4—C14	1.142 (3)	C11—H11A	0.9600
C1—C6	1.405 (3)	C11—H11B	0.9600
C1—C2	1.413 (3)	C11—H11C	0.9600
C1—C7	1.444 (3)	C12—H12A	0.9600
C2—C3	1.404 (3)	C12—H12B	0.9600
C3—C4	1.367 (4)	C12—H12C	0.9600
C3—H3	0.9300		
			
N3—Zn1—N4	107.10 (10)	N1—C7—C1	128.3 (2)
N3—Zn1—O1	112.62 (9)	N1—C7—H7	115.9
N4—Zn1—O1	110.76 (9)	C1—C7—H7	115.9
N3—Zn1—N1	115.61 (9)	N1—C8—C9	111.1 (2)
N4—Zn1—N1	112.86 (10)	N1—C8—H8A	109.4
O1—Zn1—N1	97.80 (8)	C9—C8—H8A	109.4
C2—O1—Zn1	123.25 (14)	N1—C8—H8B	109.4
C7—N1—C8	117.3 (2)	C9—C8—H8B	109.4
C7—N1—Zn1	120.38 (16)	H8A—C8—H8B	108.0
C8—N1—Zn1	122.30 (18)	C10—C9—C8	110.9 (2)
C11—N2—C12	111.5 (2)	C10—C9—H9A	109.5
C11—N2—C10	112.6 (2)	C8—C9—H9A	109.5
C12—N2—C10	109.6 (2)	C10—C9—H9B	109.5
C11—N2—H2	108.9 (16)	C8—C9—H9B	109.5
C12—N2—H2	107.9 (16)	H9A—C9—H9B	108.1
C10—N2—H2	106.1 (16)	N2—C10—C9	113.16 (19)
C13—N3—Zn1	174.8 (2)	N2—C10—H10A	108.9
C14—N4—Zn1	169.0 (2)	C9—C10—H10A	108.9
C6—C1—C2	118.8 (2)	N2—C10—H10B	108.9
C6—C1—C7	115.3 (2)	C9—C10—H10B	108.9
C2—C1—C7	125.8 (2)	H10A—C10—H10B	107.8
O1—C2—C3	118.9 (2)	N2—C11—H11A	109.5
O1—C2—C1	124.3 (2)	N2—C11—H11B	109.5
C3—C2—C1	116.8 (2)	H11A—C11—H11B	109.5
C4—C3—C2	122.7 (2)	N2—C11—H11C	109.5
C4—C3—H3	118.6	H11A—C11—H11C	109.5
C2—C3—H3	118.6	H11B—C11—H11C	109.5
C3—C4—C5	120.6 (3)	N2—C12—H12A	109.5
C3—C4—H4	119.7	N2—C12—H12B	109.5
C5—C4—H4	119.7	H12A—C12—H12B	109.5
C6—C5—C4	118.6 (3)	N2—C12—H12C	109.5
C6—C5—H5	120.7	H12A—C12—H12C	109.5
C4—C5—H5	120.7	H12B—C12—H12C	109.5
C5—C6—C1	122.5 (3)	N3—C13—S1	178.9 (2)
C5—C6—H6	118.7	N4—C14—S2	178.3 (3)
C1—C6—H6	118.7		

### Hydrogen-bond geometry (Å, °)

**Table:**

*D*—H···*A*	*D*—H	H···*A*	*D*···*A*	*D*—H···*A*
N2—H2···O1^i^	0.89 (1)	1.86 (1)	2.737 (2)	170 (2)

Symmetry codes: (i) *x*−1/2, −*y*+1/2, *z*−1/2.
